# Leisure‐time physical activity and participation in organized sports: Changes from 1985 to 2014 in Finland and Norway

**DOI:** 10.1111/sms.13431

**Published:** 2019-04-29

**Authors:** Frida K. S. Mathisen, Sami Kokko, Jorma Tynjälä, Torbjørn Torsheim, Bente Wold

**Affiliations:** ^1^ Department of Health Promotion and Development University of Bergen Bergen Norway; ^2^ Faculty of Sport and Health Sciences, Research Center for Health Promotion University of Jyväskylä Jyväskylä Finland; ^3^ Department of Psychosocial Science University of Bergen Bergen Norway

**Keywords:** adolescent, exercise, HBSC, member, time change, youth

## Abstract

Participation in organized sports is a popular and important part of the lives of children and adolescents and is associated with improved psychological and social health, as well as an increased likelihood of meeting physical activity (PA) recommendations. Changes in modern society, including increased car ownership and use of technology and electronic media, have led to an additional focus on the importance of health‐enhancing PA among children and adolescents. The aim of this article was to study the secular changes in self‐reports of participation in organized sports clubs and leisure‐time vigorous physical activity (LVPA), and whether the relationship between participation in organized sports clubs and LVPA has changed from 1985 to 2014. Questionnaire data were collected in two cross‐sectional samples of Finnish and Norwegian 11‐, 13‐ and 15‐year‐olds in 1985/1986 (n = 7137) and 2014 (n = 9218). Overall, participation in organized sports clubs and level of LVPA appears to have changed in the same direction in the two Nordic countries. The proportion of 11‐year‐olds reporting to be participants in organized sports clubs increased from 1985/1986 to 2014. There was an overall increase in self‐reported LVPA. The association between participation in sports clubs and LVPA was stronger in 2014 than in 1985/1986. The findings indicated subgroup differences, in particular with regard to a steeper increase in LVPA and participation in sports clubs among Finnish girls. We suggest that attention should be given to the role of organized sports to better understand secular changes in PA.

## 1 INTRODUCTION

Increased car ownership and the use of electronic media and technology have raised concerns about increases in sedentary behavior leading to a decrease in health‐promoting physical activity among children and adolescents.^1^ Physical activity (PA) is associated with numerous health benefits in school‐aged children and adolescents and appears to follow a dose‐response relationship.^2^ The global recommendation for health‐enhancing physical activity for children and adolescents is 60 minutes of moderate to vigorous physical activity (MVPA) a day. Vigorous physical activity (VPA) is recommended at least three times per week.^2^ VPA is a subdomain of physical activity and connected to young people's recreational hobbies and sports outside school.^3^

Recent reviews on temporal trends in PA levels among children and adolescents have documented inconsistent trends in different contexts of PA,^4^, ^5^ although the evidence for a decline in activity in clearly defined contexts, such as active transport, is more consistent. The variation in methodology and measurements makes it difficult to present a coherent picture of the development in PA. A recent report from the Health Behaviour in School‐aged Children (HBSC) study^3^ found an increase from 2002 to 2014 in prevalence of self‐reported leisure‐time VPA (LVPA) among 11‐, 13‐, and 15‐year‐olds in several countries, including Norway. In Finland however, there was a decrease in LVPA among boys over this time period. Others have found, based on objective measures, a decrease in overall PA among Norwegian children and adolescents over a similar time period.^6^ Trends in young people's PA can be linked to macro‐environmental and demographic changes, typical of developed countries, such as less space for play in urban contexts, increased concerns about safety, changes in the roles of significant others,^7^ and innovations within the sports and exercise domain.

In a modern lifestyle, time after school has an important potential for physical activity in the lives of children and adolescents. Structured leisure‐time activities might contribute to higher levels of intrinsic motivation and positive youth development^8^ and are associated with better physical and mental health among adolescents.^9^ In contrast to other Western countries, like the United States and UK, organized sports in northern Europe and the Nordic countries in particular is strongly related to time after school, and there has been little or no collaboration between school and sports clubs.^10^ It is estimated that 88% in Finland^11^ and 70%‐80% of all children in Norway^12^ at some point during their childhood or adolescence are members of a sports club or team. Organized sports provide structures for play, social interaction, and the development of skills. More than other forms of leisure‐time PA for children and adolescents, participation in organized sports clubs is associated with improved psychological and social health,^13^ increased likelihood of meeting PA recommendations,^14^, ^15^, ^16^ and higher levels of VPA.^16^, ^17^, ^18^ Furthermore, membership in sports clubs during adolescence predicts higher levels of leisure‐time PA in adulthood.^19^, ^20^

Previous trend studies in four different European countries showed an increase in organized sports participation among children and adolescents.^10^, ^21^, ^22^, ^23^ Participation in sports clubs has increased in Finland over the last ten and twenty years, as the proportion of participants was 48% for boys and 44% for girls in 2010, and 46% for boys and 34% for girls in 1990.^24^ In Norway, studies have found relatively stable numbers of membership in organized sports clubs from 1992 to 2010.^25^ In 1987, the Norwegian Provisions on Children's Sports was passed, stating that children's sports, up to the age of 13, should be based on versatility and play.^26^ This included limiting early specialization, only allowing children under the age of 10 to participate in competitions in their local sports club or local community and banning schedules and lists of results for children under the age of 12.^12^ In 2007, the provision was revised, with a new section focusing on children's rights in sports and a simplified language.^26^

However, participation in organized sports decreased from 1992 to 2010 among older adolescents in Norway.^25^ The decrease in participation among older adolescents may relate to an increased focus on specialization and pressure both within organized sports and from school.^27^ A Swedish study found that from 1974 to 1995, adolescents participated less in traditional team sports activities, while interest and participation in keep‐fit activities outside traditional sports clubs increased. The authors suggest that this could partly be explained by a larger range of activities offered in 1995 compared to 1974 and by increased body and health awareness.^23^

New, informal sports, such as keep‐fit activities, jogging, strength training, cross fit, hiking, and skateboarding, have gained increasing popularity among adolescents.^23^, ^28^ Informal sports do not require adolescents to be part of organized official sports clubs,^28^ but may involve high levels of LVPA. Thus, the introduction of new types of physical activities may contribute to a change in the importance of participating in organized sports clubs for adolescents’ LVPA levels. This may be even more relevant for older adolescents, as they may be more likely to choose these new types of activities than younger adolescents may, the latter being more constrained by parents and caretakers in their choice of leisure‐time activities. Previously, the importance of participation in organized sports for level of overall PA and LVPA has been documented in cross‐sectional studies,^14^, ^17^, ^29^ and changes over time have not been identified.^18^ To this end, Ekelund et al^5^ called for repeated cross‐sectional surveys of population‐representative samples in order to properly examine the magnitude and direction of recent changes in PA levels and sports participation of adolescents.^5^

The aim of this study was to examine changes from 1985 to 2014 in self‐reported participation in organized sports clubs and LVPA among Finnish and Norwegian children and adolescents and to what extent the association between self‐reports of participation in organized sports clubs and adolescents’ LVPA levels has changed over this time in the two countries.

## 2 METHODS

### 2.1 Procedures

This study uses data from the Finnish and Norwegian contributions to the repeated cross‐sectional study, Health Behaviour in School‐aged Children (HBSC), a WHO cross‐national survey. This international study is carried out in collaboration with WHO/EURO every 4 years on nationally representative samples of 11‐, 13‐, and 15‐year‐olds.^30^ The present paper presents data collected at two time points in 1985/1986 (November‐December 1985 in Norway and February‐March 1986 in Finland) and 2013/2014 (March‐May 2014 in Finland and March 2014‐January 2015 in Norway).Other time points or HBSC countries were not included in the analysis as the item on participation in organized sports clubs was not present in any of the other national HBSC surveys in both 1985 and 2014.

Informed passive consent, including, voluntary participation, possibility to withdraw at any time without specific reason and full anonymity throughout the study, was obtained from the pupils’ primary guardians and all pupils gave voluntary informed consent. In accordance with the international protocol of the HBSC study, 90% of the sample in each age group fell between half a year of a mean age of 11.5, 13.5, and 15.5 years. The respondents anonymously completed the questionnaires during one lesson period at school with their teacher following a standard set of instructions. The respondents were informed about the study and that participation was voluntary. In 1985/1986, the questionnaire was paper based in both countries, as was the questionnaire in Finland in 2014, while the schools could choose a paper‐based or a web‐based questionnaire in Norway. At both time points, the Data Protection Official for Research at the Norwegian Center for Research Data assured that the study complied with the ethical requirements for privacy and confidentiality. The Finnish HBSC study was approved by the Finnish Teachers Trade Union and the Finnish National Agency for Education when the survey was collected the first time in 1986 and the procedure has been the same since. Data collection has followed the existing ethical guidelines with passive consent procedure.

### 2.2 Sample

A total of 16 482 (Table 1) 11‐, 13‐, and 15‐year‐olds participated in the surveys in 1985/1986 and 2014 (student response rates among participating schools were 89% and 85% in Finland and 91% and 92% in Norway, respectively). In Finland, the samples were chosen from the Finnish school register by using a special sampling program. The sample frame was the number of pupils at each class level. Schools were selected using a cluster sampling method that took the size of the schools (PPS, probability proportionate to size) into account. Inside a selected school, one class was randomly selected. In Norway, school classes were used as primary sampling units, with a standard cluster sampling procedure based on a graphical stratified list and sequential selection from a randomized starting point. If a school agreed to participate, all pupils from one class per age group were selected.

**Table 1:** Sample size, mean age, and standard deviation by country, age group, gender, and survey year

	1985/1986			2014		
	n	Mean age	SD	n	Mean age	SD
Finland						
11‐year‐old boys	578	11.7	0.29	963	11.8	0.28
11‐year‐old girls	589	11.6	0.29	1020	11.8	0.27
13‐year‐old boys	465	13.6	0.30	943	13.8	0.30
13‐year‐old girls	461	13.6	0.30	944	13.8	0.30
15‐year‐old boys	543	15.7	0.31	956	15.8	0.31
15‐year‐old girls	546	15.6	0.29	1009	15.8	0.29
Norway						
11‐year‐old boys	720	11.5	0.30	689	11.6	0.32
11‐year‐old girls	642	11.4	0.29	700	11.6	0.31
13‐year‐old boys	612	13.4	0.30	505	13.6	0.31
13‐year‐old girls	690	13.4	0.30	540	13.6	0.32
15‐year‐old boys	667	15.5	0.30	454	15.6	0.29
15‐year‐old girls	624	15.4	0.30	495	15.5	0.30

### 2.3 Measures

Two self‐reported questions regarding the respondents’ LVPA and participation in organized sports clubs were used as the basis of our analysis, as these questions were identical at both time points and included in the questionnaires for all age groups. LVPA was measured with the item “OUTSIDE SCHOOL HOURS: How often do you usually exercise in your free time so much that you get out of breath or sweat?”. The item had seven response categories (coding given in parenthesis): Every day (7), 4‐6 times a week (5), 2‐3 times a week (2.5), Once a week (1), Once a month (0.25), Less than once a month (0), or Never (0).

This LVPA item has previously been assessed and been found to have acceptable to good reliability in an Australian sample^31^ and overall good reliability in a Norwegian sample aged 13‐18 years (intraclass correlations, 0.59‐0.87).^29^ In terms of validity, the item has shown statistically significant correlations (*r* = 0.39) with physical fitness (maximal oxygen uptake)^32^ and partial validity, showing higher scores on a 20‐m shuttle run test for those who reported higher activity levels than others.

Participation in organized sports clubs was measured using a single question: “Are you a member of a sports club?”, with response categories being No (1), Yes, I train in a sports club (2), and Yes, but I don't attend training sessions (3). A small number of respondents indicated that they were members of a sports club, but did not attend training sessions (Finland: n = 313 in 1985/1986 and n = 183 in 2014, Norway: n = 258 in 1985/1986 and n = 76 in 2014). Analyses performed with the non‐attending participants included in the study showed only minor, nonsignificant differences from the active participant group (results not shown). To interpret the results more precisely and reflect upon the differences between non‐participants and participants in sports clubs, we excluded the 830 passive members from further analysis and used a dichotomized version of the item (No = 0, Yes, I train in a sports club = 1).

### 2.4 Analysis

We performed the statistical analysis using IBM SPSS Statistics version 24. To analyze secular changes and gender differences in participation in organized sports clubs and level of LVPA, we used Pearson chi‐square and independent sample *t* test. Cohen's *d* was calculated to produce effect sizes by using calculator at http://www.psychometrica.de.

The primary dependent variable in the current analysis was LVPA. Five discrete independent factors were included as follows: country, gender, survey year, participation in organized sports clubs, and age group. To account for classroom effects appropriately, all models were specified as two‐level regression models, modeling a random intercept for school class as the primary sampling unit. A full linear mixed model including all main and interactive effects was estimated using restricted maximum likelihood. Omnibus significance test for each independent factor was assessed by means of type III *F* tests using Satterthwaite corrected degrees of freedom. For the purpose of interpreting findings, estimated marginal means were estimated, with simple effects of survey year by country, participation group, gender, and age group.

## 3 RESULTS

Table 2 shows the proportion of respondents reporting to be participants in sports clubs by gender, country, age, and year. Compared with 1985/1986, more 11‐year‐old girls and boys reported participation in organized sports clubs in 2014, with small to medium effect sizes. The difference was largest among Finnish 11‐year‐old girls, with an increase of 20 percentage points in participation. With the exception of Finnish girls, the results showed a tendency for none or reduced changes in participation rates among older adolescents. The gender differences in participation in sport clubs declined during the period, with a higher proportion of boys than girls in 1985/1986 compared to 2014, especially in Finland.

**Table 2:** Percentage of respondents participating in organized sports clubs in 1985/1986 and 2014

	Boys						Girls					
	1985/1986		2014		Time difference	Effect size	1985/1986		2014		Time difference	Effect size
	n	%	n	%	*χ* ^2^	*d*	n	%	n	%	*χ* ^2^	*d*
Finland												
11‐year‐olds	279	53	555	59	5.74*	0.13	186	35	533	54	52.89**	0.38
13‐year‐olds	194	46	414	46	0.01	0.02	143	34	439	48	23.08**	0.27
15‐year‐olds	189	39	325	36	1.34	0.06	130	27	364	38	15.15**	0.21
Norway												
11‐year‐olds	470	70	425	78	10.39**	0.19	356	60	425	72	19.11**	0.26
13‐year‐olds	360	63	265	66	1.20	0.07	384	59	281	63	2.13	0.09
15‐year‐olds	374	62	206	55	4.35*	0.13	300	53	196	47	4.10*	0.13

Note

Gender differences in 1985/1986 Finland: 11 y (*χ* ^2^ = 36.46, *P* < 0.001, *d* = 0.38), 13 y (*χ* ^2^ = 12.11, *P* = 0.001, *d* = 0.24), 15 y (*χ* ^2 ^= 13.86, *P* = 0.008, *d* = 0.24). Gender differences in 2014 Finland: 11 y (*χ* ^2^ = 5.59, *P* = 0.018, *d* = 0.11), 13 y (*χ* ^2 ^= 1.28, ns), 15 y (*χ* ^2^ = 0.98, ns). Gender differences in 1985/1986 Norway: 11 y (*χ* ^2^ = 12.26, *P* < 0.001, *d* = 0.20), 13 y (*χ* ^2^ = 2.26, ns), 15 y (*χ* ^2^ = 8.90, *P* = 0.003, *d* = 0.18). Gender differences in 2014 Norway: 11 y (*χ* ^2^ = 4.66, *P* = 0.031, *d* = 0.13), 13 y (*χ* ^2^ = 0.97, ns), 15 y (*χ* ^2^ = 5.60, *P* = 0.018, *d* = 0.17).

*χ* ^2^, Pearson chi‐square.

* *P* < 0.05,

** *P* ≤ 0.001.

In general, the mean levels of LVPA were higher in 2014 than in 1985/1986 (Table 3). Among girls, the effect sizes were medium to large, amounting to more than one time LVPA more per week. Among boys, the differences were small to medium, and no statistically significant time difference in LVPA was detected in the two oldest age groups in Finland. In line with the findings on self‐reported participation in sports clubs, gender differences in LVPA declined in Finland and were non‐existent in the two oldest age groups in 2014. In Norway, however, boys reported a higher level of LVPA than girls at both time points.

**Table 3:** Mean levels, standard deviations, and t test values of times per week in leisure‐time vigorous physical activity in 1985/1986 and 2014

	Boys								Girls							
	1985/1986			2014			Time difference	Effect size	1985/1986			2014			Time difference	Effect size
	n	*M*	SD	n	*M*	SD	*t*	*d*	n	*M*	SD	n	*M*	SD	*t*	*d*
Finland																
11‐year‐olds	577	2.97	2.21	956	3.39	2.39	−3.45*	0.18	589	2.21	1.83	1011	3.13	2.27	−8.86*	0.43
13‐year‐olds	463	3.26	2.31	887	3.38	2.39	−0.91	0.05	461	2.25	1.88	927	3.26	2.29	−8.81*	0.47
15‐year‐olds	540	3.11	2.38	928	3.36	2.46	−1.86	0.10	546	2.19	2.00	994	3.40	2.33	−10.71*	0.55
Norway																
11‐year‐olds	718	3.22	2.10	583	3.96	2.12	−6.29*	0.35	641	2.52	1.81	627	3.49	1.96	−9.12*	0.51
13‐year‐olds	611	2.97	1.90	426	3.68	2.14	−5.49*	0.35	689	2.67	1.87	476	3.36	1.94	−6.01*	0.36
15‐year‐olds	666	3.03	2.11	393	3.80	2.37	−5.34*	0.34	623	2.61	1.95	446	3.37	2.04	−6.17*	0.38

Note

Gender differences in 1985/1986 Finland: 11 y (*t* = 6.42, *P* < 0.001, *d* = −0.38), 13 y (*t* = 7.32, *P* < 0.001, *d* = −0.48), 15 y (*t* = 6.96, *P* < 0.001, *d* = −0.42). Gender differences in 2014 Finland: 11 y (*t* = 2.51, *P* = 0.012, *d* = −0.11), 13 y (*t* = 1.08, ns), 15 y (*t* = −0.34, ns). Gender differences in 1985/1986 Norway: 11 y (*t* = 6.55, *P* < 0.001, *d* = 0.36), 13 y (*t* = 2.86, *P* = 0.004, *d* = 0.15), 15 y (*t* = 3.74, *P* < 0.001, *d* = 0.21). Gender differences in 2014 Norway: 11 y (*t* = 4.02, *P* < 0.001, *d* = 0.23), 13 y (*t* = 2.36, *P* = 0.02, *d* = 0.16), 15 y (*t* = 2.82, *P* = 0.005, *d* = 0.19).

* *P* < 0.001

**Figure 1:**
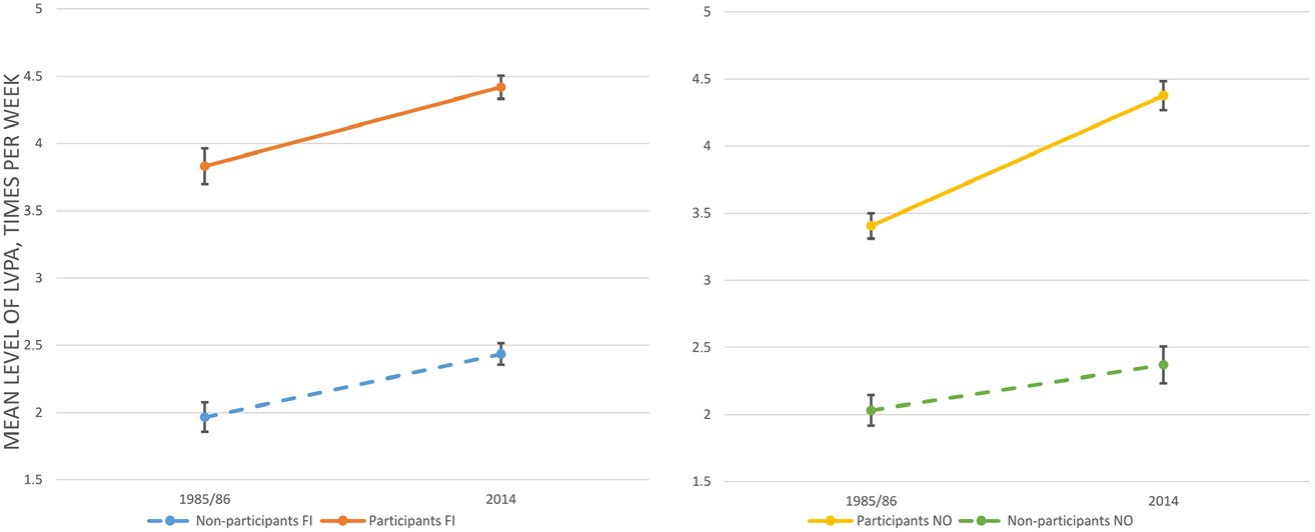
Mean level of weekly LVPA for participants and non‐participants in organized sports in 1985 and 2014 in Finland (left) and Norway (right)

**Figure 2:**
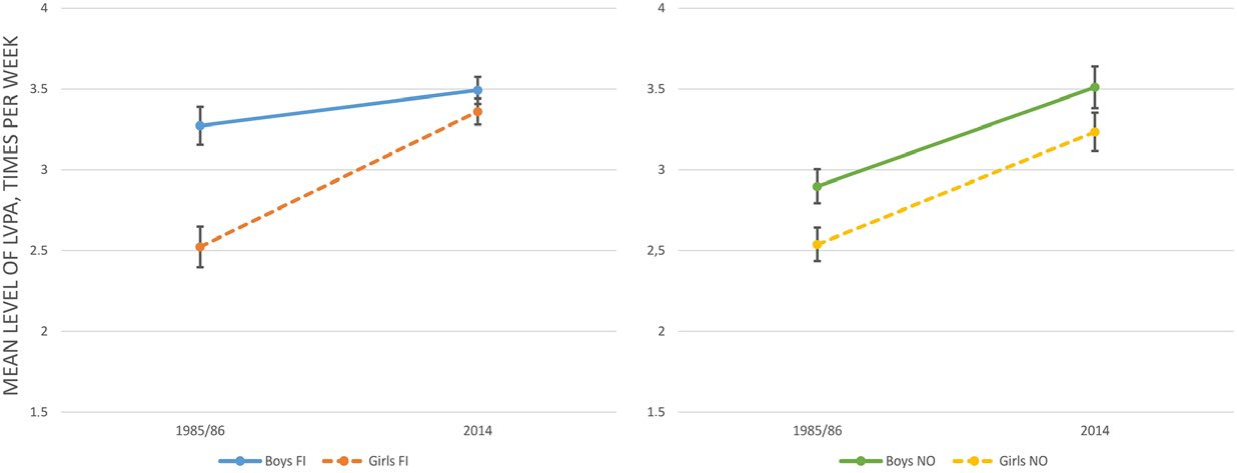
Mean level of weekly LVPA for boys and girls in 1985 and 2014 in Finland (left) and Norway (right)

Linear mixed model analysis was performed to establish whether LVPA differed according to country, gender, age group, participation in organized sports clubs, and survey year (Table 4). There were significant main effects of all independent variables. As shown in Table 5, the activity levels were generally higher in 2014, among boys, and among those in organized sports clubs. Inspection of the means in Table 5 reveals that the effects of age and country were negligible, although statistically significant. With regard to difference over time, there was a two‐way interaction (Table 4) between survey year and participation in sports clubs on LVPA, indicating that the effect of participation in organized sports clubs differed across survey year. Table 5 shows that this difference concerns a stronger association between participation in sports clubs and LVPA in 2014 compared to 1985/1986, that is in 2014, participants in sports clubs reported higher levels of LVPA compared to non‐participants than was the case in 1985/1986. There was a two‐way interaction effect of survey year and gender. The means depicted in Table 3 suggest that this effect is due to the relatively steeper increase over time in LVPA among girls compared to boys. Figure 1 illustrates the significant three‐way interaction effect between participation in organized sports clubs, country, and survey year on LVPA shown in Table 4. In Finland, the increase in the association between participation and LVPA over time was less pronounced than in Norway. Figure 2 illustrates the three‐way interaction between gender, country, and survey year. From 1985/1986 to 2014, the effect of gender on LVPA changed more in Finland than in Norway, indicated by a steeper increase in activity among Finnish girls.

**Table 4:** Univariate analysis of variance testing interaction effects of gender, age group, participation in organized sports clubs, and survey year on times per week in leisure‐time vigorous physical activity (N = 14 760)

Variable	Numerator *df*	Denominator *df*	*F*	*P*
Intercept	1	838.19	20623.90	<0.001
Survey year (Y)	1	838.19	187.44	<0.001
Gender (G)	1	14599.62	116.55	<0.001
Age group (A)	2	866.92	16.42	<0.001
Participation in organized sports clubs (P)	1	14626.74	2588.11	<0.001
Country (C)	1	838.19	7.28	0.007
Y * G	1	14599.62	24.88	<0.001
Y * A	2	866.92	2.10	0.123
Y * P	1	14626.74	27.94	<0.001
Y * C	1	838.19	2.13	0.145
G * A	2	14596.74	1.18	0.307
G * P	1	14696.66	5.94	0.015
G * C	1	14599.62	3.05	0.081
A * P	2	14629.69	49.88	<0.001
A * C	2	866.92	6.74	0.001
P * C	1	14626.74	10.84	0.001
Y * G * A	2	14596.74	0.31	0.733
Y * G * P	1	14696.66	2.55	0.110
Y * G * C	1	14599.62	14.38	<0.001
Y * A * P	2	14629.69	1.41	0.245
Y * A * C	2	866.92	0.32	0.725
Y * P * C	1	14626.74	13.05	<0.001
G * A * P	2	14696.65	0.83	0.435
G * A * C	2	14596.74	1.99	0.137
G * P * C	1	14696.66	0.15	0.699
A * P * C	2	14629.69	4.16	0.016
Y * G * A * P	2	14696.65	0.83	0.435
Y * G * A * C	2	14596.74	1.41	0.243
Y * G * P * C	1	14696.66	1.88	0.170
Y * A * P * C	2	14629.69	2.17	0.114
G * A * P * C	2	14696.65	0.88	0.415
Y * G * A * P * C	2	14696.65	0.08	0.924

**Table 5:** Means, standard error, and univariate tests for the simple effects of survey year on times per week in leisure‐time vigorous physical activity for boys and girls within each age group and country for non‐participants and participants in organized sports clubs

	Gender	1985/1986		2014		Numerator* df*	Denominator* df*	*F*	*P*
		*M*	SE	*M*	SE				
Non‐participants in organized sports clubs (n = 7025)									
Finland									
11‐year‐olds	Boys	2.23	0.13	2.67	0.11	1	5496.79	6.57	0.010
	Girls	1.74	0.12	2.35	0.10	1	3705.88	16.53	<0.001
13‐year‐olds	Boys	2.40	0.14	2.33	0.10	1	4354.57	0.15	0.701
	Girls	1.77	0.13	2.22	0.10	1	3802.38	7.47	0.006
15‐year‐olds	Boys	2.13	0.13	2.55	0.09	1	3519.73	7.80	0.005
	Girls	1.53	0.12	2.49	0.09	1	2994.47	43.56	<0.001
Norway									
11‐year‐olds	Boys	2.41	0.14	2.67	0.19	1	10305.13	1.22	0.269
	Girls	1.90	0.14	2.32	0.16	1	8741.30	4.01	0.045
13‐year‐olds	Boys	2.14	0.14	2.32	0.18	1	7515.48	0.64	0.423
	Girls	1.89	0.13	2.20	0.16	1	5819.19	2.35	0.126
15‐year‐olds	Boys	1.92	0.14	2.39	0.16	1	6269.18	5.03	0.025
	Girls	1.93	0.13	2.31	0.14	1	4963.34	4.09	0.043
Participants in organized sports clubs (n = 7735)									
Finland									
11‐year‐olds	Boys	3.67	0.13	3.93	0.09	1	4188.92	2.78	0.095
	Girls	2.92	0.15	3.84	0.09	1	5405.56	26.61	<0.001
13‐year‐olds	Boys	4.39	0.15	4.66	0.10	1	5111.43	2.12	0.145
	Girls	3.35	0.17	4.36	0.10	1	6062.40	25.61	<0.001
15‐year‐olds	Boys	4.82	0.15	4.82	0.12	1	6173.68	0.00	0.992
	Girls	3.84	0.18	4.91	0.11	1	7108.10	25.74	<0.001
Norway									
11‐year‐olds	Boys	3.62	0.10	4.33	0.10	1	3484.50	24.86	<0.001
	Girls	2.97	0.11	3.95	0.10	1	3993.29	40.83	<0.001
13‐year‐olds	Boys	3.47	0.11	4.43	0.13	1	3800.69	31.63	<0.001
	Girls	3.24	0.11	4.05	0.13	1	3423.20	23.21	<0.001
15‐year‐olds	Boys	3.84	0.11	4.92	0.15	1	4410.53	35.24	<0.001
	Girls	3.31	0.12	4.59	0.15	1	4765.40	44.12	<0.001

## 4 DISCUSSION

Overall, participation in organized sports clubs and level of LVPA appears to have changed in the same direction in the two Nordic countries. The proportion of 11‐year‐olds reporting to be participants in organized sports clubs increased from 1985/1986 to 2014. There was an overall increase in self‐reported LVPA. The association between participation in sports clubs and LVPA was stronger in 2014 than in 1985/1986. The findings indicated subgroup differences, in particular with regard to a steeper increase in LVPA and participation in sports clubs among Finnish girls.

The increase in participation among the 11‐year‐olds is consistent with the results in other European studies.^10^, ^21^, ^22^ A Norwegian study has indicated that children in 2006 started participating in sports at an earlier age than before.^12^ This is also a prevalent trend in Finland, as the involvement in organized sports starts younger today, on average at the age of six. One explanation can be that the strong public debate concerning low levels of PA among children and adolescents has made parents regard sports participation as a major solution for PA promotion among their children.^11^

Fewer 15‐year‐olds in Norway reported participating in organized sports clubs in 2014 compared to 1985/1986. In another Norwegian study, more respondents dropped out of organized sports by the age of 15 in 2010 than in 1992.^25^ The observed change among 11‐ and 15‐year‐olds might be based on earlier recruitment into organized sports, which may result in earlier dropout. Previous research has described similar changes, suggesting that involvement in organized sports has trickled down to the youngest adolescents.^21^ However, although the observed changes are statistically significant, the effect sizes related to these changes are small to medium.

The age‐related pattern of increased involvement among 11‐year‐olds and decreased involvement among 15‐year‐olds might also be linked to the introduction of the Provision on Children's Sports in Norway. The provision might have contributed to a greater recruitment of younger children with its focus on mastery and play, and not skills. However, from the age of 13 years, the Norwegian guidelines for youth sports allow for more competition and increasing specialization, which can be perceived as undesirable for the adolescents.^25^ Such negative reactions to increased competitiveness were observed in a Norwegian study based on self‐reported data from 14‐16‐year‐olds in 2006. The study revealed that the most common reasons why participants had left sports clubs were that the demands for improved achievement were too high.^27^ In a similar vein, Crane and Temple's^33^ systematic review identified participants’ perceptions of their physical or sport competence as a prominent reason for dropout from organized sports.

While participation among boys in Finland and Norway seem to change in a similar way, the highest increase was among Finnish girls, as reflected in an increase of 20 percentage points among 11‐year‐olds. This finding support similar results from other studies in Finland on girls’ participation in sports clubs.^11^, ^24^ The observed increase may be explained by the development within sports clubs in Finland. During the last 20‐30 years, a wider variation of different activities that attract girls more than boys, such as dancing and cheerleading, have been made available within sports clubs.^11^ It is important to notice that the significant increase in participation among Finnish girls comes from a low proportion of participants in 1985/1986, thereby having greater potential for an increase.

At both time points, the proportion of participants in organized sports clubs were higher in Norway compared to Finland. Previous research has pointed toward favorable socioeconomic conditions, high availability for sporting facilities, a well‐established voluntary sports club sector, high levels of parental involvement, and growth in individual and social prosperity during the 1990s as reasons for the high levels and increases in participation in Norway.^34^

The present findings suggest that self‐reported LVPA among Finnish and Norwegian children and adolescents increased from 1985/1986 to 2014. Similar changes in the prevalence of LVPA from 2002 to 2014 were found among 15‐year‐olds in several other countries within the HBSC study.^3^ Moreover, these findings are consistent with previous reviews,^4^, ^5^ and contradict the popular notion that children and adolescents’ level of physical activity has declined over the past decades.

Participants in organized sports clubs reported higher level of LVPA compared to non‐participants at both time points. Several other studies have also documented a positive relationship between sport participation and VPA.^16^, ^17^, ^18^, ^35^ The difference in level of LVPA between participants and non‐participants was higher in 2014 than in 1985/1986, shown in Figure 1. Possibly, the larger difference in 2014 points to changes within organized sport as a driving force for changes in overall LVPA. For all age groups, the level of commitment when participating in organized sports clubs might have increased, resulting in that more of the LVPA among children and adolescents is carried out in organized structures such as sports clubs. Research on secular changes in Norway from 1992 to 2010 found an increase in the reported number of times per week adolescents participated in organized sports.^25^ Increased commitment and frequency might imply that the importance of participation in organized sports clubs for higher levels of LVPA has increased from 1985/1986 to 2014. Macro‐environmental and demographic changes such as less space for spontaneous PA and increased preferences for safe and structured activities might have influenced children and adolescents PA behavior. In addition, increased parental involvement in their children's participation in sports, which is considered a generational change,^37^ may have led to the observed increase in the association between participation and level of LVPA among children and adolescents as parents encourage and arrange for participation in sports clubs.

For the 15‐year‐olds in Norway, the change can be related to selection as we found a decrease in the proportion of participants in organized sports clubs in this age group. Those 15‐year‐olds who participate in organized sports clubs might invest more time and energy in activities related to this sport, resulting in a stronger association between participation in organized sports clubs and LVPA in 2014 compared to 1985/1986.

In Finland, a substantial increase in LVPA was observed also among non‐participating girls, perhaps related to secular changes in the engagement in keep‐fit activities outside organized structures or within new arenas, like commercial fitness centers. Previous research on secular changes from 1974 to 1995 in Sweden found a decrease in participation in traditional team sports and that activities outside organized sports clubs had increased in popularity. Increased focus on a healthy lifestyle and participation in keep‐fit activities was proposed as a possible contribution to increased levels of physical activity outside organized structures.^23^ As we do not measure how much of the self‐reported LVPA that is related to activities within organized sports clubs, it is possible that the increased focus on a healthy lifestyle, keep‐fit activities, and informal sports^23^, ^28^ also contributed to the increase in LVPA among those who participate in organized sports clubs.

This study is not without limitations. As the study is cross‐sectional, the causal direction of the relationship between participation in organized sports clubs and frequency of LVPA cannot be established. Participation in organized sports clubs can lead to higher levels of LVPA, at the same time as high levels of LVPA can lead to participation in organized sports clubs. Further, self‐reported activity might be affected by the respondents’ desire to provide socially desirable answers, or by recall bias.^38^ Several of these biases could be avoided through objective measures of physical activity. However, it is difficult to find comparable objective measures, mainly because our data go back to 1985, a time when objective measures of physical activity were not commonly used within this research field. In Norway, the comparability of the data from 1985/1986 and 2014 might have been affected by seasonal variance, as the data collection in 1985 was mid‐winter, while in 2014 it was done from early spring to mid‐winter. However, we did not find significant differences between the two subsamples from spring and mid‐winter in 2014 (results not shown). A Norwegian study using objective measurements concluded that seasonal variation influenced children more than adolescents, as they found no association between season and PA level among 15‐year‐olds although this was the case among 9‐year‐olds.^39^ It is worth noticing that the geographical distribution of the population in Norway might result in seasonal variances because of the variation in climate from north to south. The variation in measurement point would however been more relevant if the measures used were related to type of activity and not frequency of LVPA. The main strengths of this study are the relatively large and population‐based samples and the comparable and repeated measurements from Finland and Norway following the HBSC protocol both time points.

## 5 PERSPECTIVES

Participation in organized sports clubs by children and adolescents is a common and popular leisure‐time activity. Increased recruitment into organized sports has the potential to benefit the lives of children and adolescents in terms of positive youth development and both psychological and social health and gives opportunities to develop positive health behaviors that endure across the lifespan.

Our results support positive development of improved recruitment into organized sports among 11‐year‐olds in both countries. However, we suggest that more attention should be given to the possible negative development of reduced involvement among older adolescents and to the increased difference in LVPA between participants and non‐participants in organized sports in Norway. Organized sports clubs should continue to focus on preventing dropout, that is, keeping more adolescents involved in this positive setting for LVPA. In Finland, the positive development in both participation in organized sports clubs and level of LVPA among girls should be further investigated and continuously promoted.

Results of previous research on secular changes and trends in PA among children and adolescents has been inconsistent, and the need for studying specific domains within PA has been addressed.^5^ The overall levels of LVPA increased from 1985 to 2014 in this sample of 11 to 15‐year‐olds in Finland and Norway. However, the development differed between different subgroups. This adds to the importance of studying the role of participation and gender differences to better understand secular PA changes in a modern society.
